# Fetal lung elasticity changes following antenatal betamethasone administration assessed by shear-wave elastography

**DOI:** 10.3389/fped.2026.1873863

**Published:** 2026-06-24

**Authors:** Gusztav Labossa, Reka A. Vass, Tibor Ertl, Jozsef Bodis, Kalman Kovacs

**Affiliations:** 1 Department of Obstetrics and Gynecology, Medical School University of Pécs, Pécs, Hungary; 2 MTA-PTE Human Reproduction Scientific Research Group, University of Pécs, Pécs, Hungary; 3 National Laboratory on Human Reproduction, University of Pécs, Pécs, Hungary; 4 Clinical Centre, University of Pécs, Pécs, Hungary

**Keywords:** antenatal corticosteroids, betamethasone, fetal lung maturation, lung elasticity, shear-wave elastography

## Abstract

**Background:**

Antenatal corticosteroids are widely used to accelerate fetal lung maturation in pregnancies at risk of preterm birth. However, a reliable non-invasive method to directly assess the fetal lung response to therapy *in utero* is lacking. Shear-wave elastography (SWE) has emerged as a potential imaging technique for evaluating fetal lung tissue properties.

**Methods:**

This retrospective observational study included 26 pregnancies between 24 and 34 weeks of gestation. Fetal lung and liver elasticity were assessed using two-dimensional SWE before maternal betamethasone administration, 1 min after treatment, and 24 h later.

**Results:**

Fetal lung elasticity demonstrated a gestational age–dependent decrease, with significantly lower values at 32–34 weeks compared with earlier gestational ages. No significant change in lung elasticity was observed 1 min after corticosteroid administration. However, lung elasticity decreased significantly at 24 h (from 1.87 ± 0.04 kPa to 1.72 ± 0.04 kPa, *p* = 0.0005). Liver elasticity remained unchanged throughout the observation period.

**Conclusion:**

Fetal lung elasticity measured by SWE decreases within 24 h following antenatal betamethasone administration. These findings suggest that SWE may detect corticosteroid-associated changes in fetal lung mechanical properties *in utero*. Further studies are needed to determine the clinical relevance of this approach.

## 1 Introduction

Antenatal corticosteroid administration remains one of the most effective interventions for reducing neonatal respiratory morbidity and mortality in pregnancies at risk of preterm birth. Numerous studies have demonstrated that antenatal exposure to betamethasone or dexamethasone accelerates fetal lung maturation through stimulation of surfactant production and structural lung development, thereby significantly decreasing the incidence of neonatal respiratory distress syndrome (RDS) and improving survival of preterm infants (1–4). Consequently, current international guidelines recommend antenatal corticosteroid therapy between 24 and 34 weeks of gestation when preterm delivery is anticipated (4).

In routine clinical practice, corticosteroids are administered intramuscularly to the mother and reach the fetus through transplacental transfer. Although this approach is effective, fetal drug exposure may vary depending on maternal metabolism, placental function, and comorbid conditions such as diabetes or hypertensive disorders of pregnancy (5, 6). Despite the widespread use of antenatal corticosteroids, clinicians currently lack a reliable, non-invasive method to directly assess the fetal lung response to therapy *in utero*. The biological effect of corticosteroids occurs within a limited therapeutic window, and both insufficient exposure and unnecessary treatment may have clinical consequences (7–9). At present, the effectiveness of antenatal corticosteroid therapy is inferred indirectly from gestational age and clinical context rather than measured through fetal-specific physiological changes. Therefore, the development of imaging techniques capable of monitoring fetal lung maturation before birth remains an important goal in perinatal medicine.

Shear-wave elastography (SWE) is an ultrasound-based imaging technique that quantitatively measures tissue stiffness using acoustic radiation force impulses (10). Recent studies have demonstrated the feasibility of measuring fetal lung elasticity with SWE and have shown that lung stiffness decreases progressively with advancing gestational age, suggesting that elastographic measurements may reflect structural lung maturation (11–15). However, existing studies have largely focused on cross-sectional assessments, and dynamic *in vivo* changes in fetal lung mechanical properties following antenatal corticosteroid administration have not been systematically characterized.

The ability to detect corticosteroid-induced changes in fetal lung elasticity could provide a novel approach to evaluating the biological response to antenatal steroid therapy. Such a method may offer a non-invasive, real-time means of assessing fetal lung maturation and could contribute to optimizing the timing and use of corticosteroid treatment in high-risk pregnancies.

In the present study, we therefore investigated longitudinal changes in fetal lung elasticity using two-dimensional shear-wave elastography before and after maternal antenatal betamethasone administration. By performing measurements immediately after treatment and again 24 h later, and by including fetal liver elasticity as an internal reference tissue, we aimed to determine whether SWE can detect corticosteroid-associated changes in fetal lung tissue mechanics *in vivo*.

## 2 Methods

### 2.1 Study design and population

Elastography measurements were performed as part of routine clinical assessment and subsequently analyzed retrospectively.The study was conducted at the Department of Obstetrics and Gynecology, University of Pécs, Hungary, between August 2021 and December 2023. The study was approved by the Regional and Local Research Ethics Committee of the University of Pécs (approval numbers: PTE KK 7072-2018 and PTE KK 10086-PTE2025). Written informed consent was obtained from all participants prior to inclusion. Pregnant women at risk of preterm birth who received antenatal corticosteroid therapy according to institutional protocols were eligible for inclusion. In cases of imminent preterm labor, patients received standard care including tocolytic therapy and intramuscular betamethasone administration. Following betamethasone administration, maternal blood pressure and fetal well-being were monitored throughout the observation period, and any adverse maternal or fetal events were recorded.

### 2.2 Shear-wave elastography measurements

Fetal lung and liver elasticity were assessed using two-dimensional shear-wave elastography (2D-SWE) with a Logiq E10 ultrasound system (General Electric Company, Boston, MA, USA). SWE is an ultrasound-based technique that quantifies tissue stiffness by measuring the propagation of shear waves generated by acoustic radiation force impulses (ARFI), displayed as a real-time, color-coded elastogram superimposed on a B-mode image. Measurements were performed using the system's single-shot acquisition mode with a 2.5 MHz acoustic radiation force impulse. A circular region of interest (ROI) of approximately 5 mm in diameter was placed within the target tissue in an area demonstrating reliable shear-wave propagation, as verified by the propagation map. For each examination, three separate measurements were obtained from both the fetal lung and liver, and the mean elasticity value was calculated. Elasticity values were expressed in kilopascals (kPa). All elastography measurements were performed by a single experienced operator using a standardized acquisition protocol. To minimize bias, the operator followed predefined criteria for ROI placement and image quality assessment throughout the study.

### 2.3 Image acquisition protocol

Image acquisition was standardized across all participants. Measurements were performed at depths not exceeding 8 cm. Care was taken to apply minimal transducer pressure to the maternal abdominal wall to avoid compression artifacts. ROIs were positioned within homogeneous regions of the fetal lung and liver, avoiding the vessels, ribs, and areas with poor signal quality. ROI size was adjusted as necessary to ensure optimal measurement conditions.

### 2.4 Neonatal data collection

All newborns were examined by pediatricians immediately after birth. Examiners were blinded to prenatal elastography findings. The following neonatal data were collected: gestational age at delivery, birth weight, Apgar scores at 1 and 5 min, and overall neonatal outcome.

### 2.5 Statistical analysis

Statistical analyses were performed using SPSS version 26.0 (IBM Corp., Armonk, NY, USA) and GraphPad Prism version 9.5.1 (GraphPad Software, San Diego, CA, USA). The distribution of continuous variables was assessed using visual inspection of histograms and the Shapiro–Wilk test. Homogeneity of variances was evaluated using Levene's test. Normally distributed data are presented as mean ± standard error of the mean (SEM). Differences in fetal lung and liver elasticity across gestational age groups were analyzed using one-way analysis of variance (ANOVA) with appropriate *post hoc* comparisons. To evaluate changes in elasticity over time (before corticosteroid administration, 1 min after administration, and 24 h after administration), repeated measures analysis of variance (repeated measures ANOVA) was performed. When assumptions for repeated measures ANOVA were violated, appropriate corrections were applied. A two-tailed *p*-value of <0.05 was considered statistically significant.

## 3 Results

### 3.1 Study population

A total of 26 pregnant women at risk of preterm birth were included in the study. All participants received standard antenatal corticosteroid therapy with maternal intramuscular betamethasone.

Maternal and neonatal baseline characteristics are summarized in Table 1. The mean maternal age was 29.24 ± 1.24 years. The mean gestational age at the time of betamethasone administration was 28.54 ± 0.68 weeks, and the mean gestational age at delivery was 35.66 ± 0.76 weeks. The mean birth weight of the newborns was 2627.9 ± 182.6 g. Apgar scores were within the normal range at both 1 min (8.66 ± 0.11) and 5 min (9.71 ± 0.11). No significant adverse neonatal outcomes were observed.

**Table 1:** Clinical data of involved patients.

Characteristics	Maternal betamethasone injection (*n* = 26)
Maternal age (years)	29.24 ± 1.24
Gestational age (weeks) at the time of betamethasone injection	28.54 ± 0.68
Gestational age (weeks) at the time of birth	35.66 ± 0.761
Birthweight (gram)	2,627.91 ± 182.62
Gender of newborn	
Boy	15
Girl	11
Mode of delivery	
Spontaneous	15
Caesarian section	11
Apgar score	
1 min	8.66 ± 0.11
5 min	9.71 ± 0.11

### 3.2 Gestational Age–dependent changes in fetal elasticity

Baseline fetal lung and liver elasticity values were analyzed according to gestational age groups (23–27 weeks and 32–34 weeks). A significant gestational age–dependent change was observed in fetal lung elasticity. Specifically, lung elasticity values were significantly lower in the 32–34-week group compared with the earlier gestational age group (*p* < 0.05). In contrast, fetal liver elasticity did not show significant variation across gestational age groups. These findings indicate that decreasing lung elasticity is associated with advancing gestation, while liver elasticity remains relatively stable (Table 2).

**Table 2:** Fetal lung and liver elasticity values (kPa) and lung to liver ratio at different gestational weeks .

Organ elasticity values	23–27th weeks *n* = 11)	32–34th week (*n* = 11)
Lung (kPa)	1.94 ± 0.07	1.74 ± 0.05*
Liver (kPa)	2.48 ± 0.08	2.47 ± 0.16

Data is expressed in mean ± SEM. *p = 0.008 compared to 23–27th weeks.

### 3.3 Changes in fetal lung elasticity following betamethasone administration

**Figure 1:**
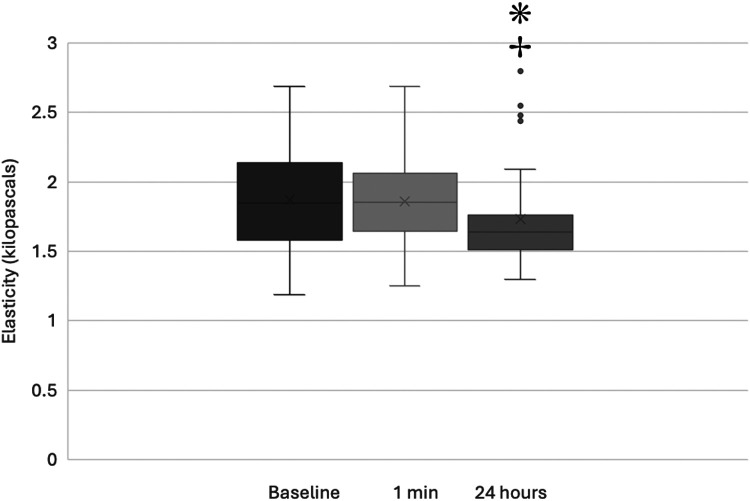
Elasticity of fetal lung after betamethasone treatment. ❊ *p* = 0.0015 1 min vs. 24 h ✢ *p* = 0.0005 before vs. 24 h.

Fetal lung and liver elasticity were measured before betamethasone administration, 1 min after administration, and 24 h after treatment. No significant change in fetal lung elasticity was observed at 1 min following corticosteroid administration compared with baseline values (*p* > 0.05). However, a significant decrease in lung elasticity was detected 24 h after treatment (*p* = 0.00005). Mean fetal lung elasticity decreased from approximately 1.87 ± 0.04 kPa at baseline to 1.72 ± 0.04 kPa at 24 h. In contrast, fetal liver elasticity remained unchanged across all time points, with no statistically significant differences observed (*p* > 0.05) (Figure 1).

### 3.4 Safety observations

Maternal blood pressure remained within physiological ranges following corticosteroid administration in all cases. Continuous fetal monitoring during the observation period did not reveal any signs of fetal compromise.

## 4 Discussion

Antenatal corticosteroid therapy remains a cornerstone in the management of pregnancies at risk of preterm birth, with well-established benefits in reducing neonatal respiratory morbidity and improving survival outcomes (16, 17). In the present study, we demonstrate that fetal lung elasticity, measured using shear-wave elastography, decreases significantly within 24 h following maternal betamethasone administration. No immediate change was observed one minute after treatment, while fetal liver elasticity remained stable across all time points. These findings suggest that shear-wave elastography is capable of detecting short-term changes in fetal lung mechanical properties following corticosteroid exposure.

The absence of a detectable change in lung elasticity immediately after corticosteroid administration, followed by a significant decrease at 24 h, supports the interpretation that elastographic changes reflect biological processes rather than acute hemodynamic or mechanical effects. Antenatal corticosteroids are known to stimulate surfactant production, enhance alveolar stability, and promote structural maturation of the fetal lung. These processes are time-dependent and consistent with the delayed elastographic response observed in our study. Importantly, the lack of change in liver elasticity supports the organ-specific nature of this response, suggesting that the observed decrease in lung elastography is not attributable to generalized fetal or systemic effects. Interestingly, the decrease in fetal lung elasticity observed 24 h after betamethasone administration resulted in values approaching those measured at baseline in the most advanced gestational age group (32–34 weeks). Although the present study was not designed to establish equivalence between corticosteroid exposure and physiological maturation, this observation is consistent with the concept that antenatal betamethasone induces changes in lung mechanical properties that parallel those occurring during advancing gestation.

Our findings are consistent with previous studies demonstrating gestational age–dependent decreases in fetal lung elasticity, reflecting progressive lung maturation during the second and third trimesters (6, 18). In this context, the reduction in lung elasticity observed after corticosteroid administration may parallel physiological maturational processes. However, it should be emphasized that elastographic measurements provide an indirect assessment of tissue mechanical properties, and therefore the observed changes should be interpreted as being consistent with, rather than definitive evidence of, lung maturation.

Previous studies have reported that antenatal corticosteroid administration may induce transient changes in fetal physiology, including alterations in fetal heart rate patterns, biophysical activity, and pulmonary perfusion (19–22). For example, increased fetal lung perfusion following maternal betamethasone administration has been demonstrated using Doppler imaging, suggesting reduced pulmonary vascular resistance (23). Additionally, temporary reductions in fetal biophysical profile scores have been described after corticosteroid exposure, primarily related to changes in fetal breathing movements and activity (24). These findings highlight the presence of short-term physiological adaptations following corticosteroid administration. In contrast, the absence of immediate elastographic changes in our study further supports the interpretation that the observed decrease in lung elasticity at 24 h reflects structural or compositional changes rather than transient functional effects.

The clinical relevance of assessing fetal lung maturation *in utero* is underscored by the limitations of current approaches. Traditional sonographic markers and echogenicity-based assessments have demonstrated limited predictive value for neonatal respiratory outcomes. Emerging imaging techniques, including quantitative ultrasound texture analysis and elastography, aim to provide more objective measures of lung development (13, 25). In this context, shear-wave elastography offers a non-invasive and quantitative method that can be integrated into routine prenatal ultrasound examinations. The ability to detect changes in fetal lung mechanical properties following corticosteroid administration may have potential implications for optimizing treatment timing and identifying variability in treatment response.

Several factors may influence fetal lung development and the response to antenatal corticosteroids, including placental function, fetal growth restriction, and maternal comorbidities. Adverse intrauterine conditions have been associated with impaired lung development and increased neonatal respiratory morbidity (26). Therefore, a technique capable of assessing fetal lung status *in vivo* may be particularly valuable in high-risk pregnancies, where standard clinical indicators may not fully reflect fetal physiological readiness for extrauterine life.

Beyond the neonatal period, lung development continues throughout infancy and childhood, and early alterations in lung structure and function may have long-term respiratory consequences (27). Although the present study focuses on short-term changes following antenatal corticosteroid exposure, improved understanding of fetal lung maturation may contribute to strategies aimed at optimizing respiratory outcomes across the life course.

The clinical relevance of assessing fetal lung properties *in utero* is further supported by postnatal imaging studies demonstrating a close relationship between lung structure, mechanics, and respiratory outcomes. Lung ultrasound has emerged as a valuable bedside tool in preterm neonates, with specific imaging patterns correlating with the severity of respiratory distress and clinical outcomes (26). In addition, intrauterine conditions such as fetal growth restriction and placental dysfunction have been shown to adversely affect lung development, highlighting the importance of sensitive prenatal assessment methods (27). Lung development is a continuous process extending beyond birth, and early alterations in lung structure and function may have long-term consequences for respiratory health (28). Furthermore, antenatal corticosteroid administration has been associated with transient changes in fetal hemodynamics, particularly in compromised pregnancies, suggesting that systemic physiological adaptations may accompany lung-specific effects (29). The 1-minute post-administration measurement was included to determine whether betamethasone administration produced any immediate effect on elastographic measurements. Although corticosteroid-induced lung maturation would not be expected to occur within such a short time interval, the absence of a measurable change at 1 min supports the interpretation that the reduction in lung elasticity observed at 24 h is unlikely to represent an acute measurement artifact or an immediate response to drug administration. This observation is consistent with previous reports demonstrating that physiological and hemodynamic effects of maternal betamethasone administration develop over hours rather than minutes following treatment (29). In this context, fetal lung elastography may provide a non-invasive approach to capturing early changes in lung tissue properties in response to antenatal therapy.

This study has several limitations. First, the retrospective design and relatively small sample size limit the generalizability of the findings. Second, neonatal respiratory outcomes were not analyzed in relation to elastographic measurements, which restricts conclusions regarding the predictive clinical value of this technique. Third, although measurement protocols were standardized, potential variability related to operator technique and fetal positioning cannot be fully excluded. Finally, elastography provides an indirect assessment of tissue properties, and further studies correlating imaging findings with functional respiratory outcomes are needed. All measurements were performed by a single operator, which may limit generalizability, although it ensured consistency in image acquisition. Accordingly, the present findings should be interpreted as proof-of-concept evidence that shear-wave elastography can detect changes in fetal lung elasticity following antenatal betamethasone administration rather than as definitive evidence of the underlying biological mechanisms or clinical effectiveness of treatment. Because fetal hemodynamic and physiological parameters were not assessed in the present study, the relative contribution of corticosteroid-induced tissue maturation and physiological adaptation to the observed decrease in lung elasticity cannot be determined.

In conclusion, fetal lung elasticity measured by shear-wave elastography decreases within 24 h following maternal antenatal betamethasone administration. These findings demonstrate that elastography may detect corticosteroid-associated changes in fetal lung mechanical properties *in vivo*. Further prospective studies incorporating neonatal respiratory outcomes are required to determine the clinical utility of this technique in perinatal practice.

## 5 Conclusion

In this study, fetal lung elasticity measured by shear-wave elastography decreased significantly within 24 h following maternal antenatal betamethasone administration. This change was not observed immediately after treatment and was not present in fetal liver tissue, suggesting a delayed and organ-specific response. As a proof-of-concept study, these findings provide preliminary evidence that shear-wave elastography can detect corticosteroid-associated changes in fetal lung elasticity *in vivo* and support further investigation of this technique in larger prospective studies. These findings indicate that shear-wave elastography may detect corticosteroid-associated changes in fetal lung mechanical properties *in utero*. As a non-invasive and quantitative imaging technique, fetal lung elastography has the potential to contribute to the assessment of fetal lung development and response to antenatal therapy. However, given the limited sample size and the absence of correlation with neonatal respiratory outcomes, further prospective studies are required to determine the clinical utility of this approach and its potential role in guiding perinatal decision-making.

## Data Availability

The raw data supporting the conclusions of this article will be made available by the authors, without undue reservation.
